# Current Surgical Techniques in the Treatment of Adult Developmental Dysplasia of the Hip

**DOI:** 10.3390/jpm13060942

**Published:** 2023-06-01

**Authors:** Anand S. Dhaliwal, Muzammil Akhtar, Daniel I. Razick, Arya Afzali, Ethan Wilson, Alexander J. Nedopil

**Affiliations:** 1 College of Medicine, Californa Northstate University, Elk Grove, CA 95757, USA; muzammil.akhtar9106@cnsu.edu (M.A.); daniel.razick10009@cnsu.edu (D.I.R.); arya.afzali7448@cnsu.edu (A.A.); ethan.wilson7202@cnsu.edu (E.W.); ajnedopil@ucdavis.edu (A.J.N.); 2 Orthopädische Klinik König-Ludwig-Haus, Lehrstuhl für Orthopädie der Universität Würzburg, 97074 Würzburg, Germany; 3 Department of Biomedical Engineering, University of California, Davis, CA 95616, USA

**Keywords:** hip dysplasia, surgical techniques, osteotomy, total hip arthroplasty, arthroscopy, hip preservation

## Abstract

The surgical protocols currently used for the treatment of developmental dysplasia of the hip (DDH) are varied, with sufficient differences in clinical outcomes that warrant a review of the role of practicing orthopedic surgeons. This paper aims to summarize the current novel techniques within the realm of surgical treatment for adult DDH, thus serving as a guide to surgeons looking to quickly familiarize themselves with available techniques. We performed computer systematic literature searches of the Embase and PubMed databases from 2010 to 2 April 2022. Study parameters as well as their respective patient reported outcomes (PROMs) were described in detail and compiled into diagrams. Two novel techniques were identified for the treatment of borderline or low-grade DDH. Six techniques which included modifications to the Bernese periacetabular osteotomy (PAO) were identified for the treatment of symptomatic DDH. Three techniques which include combinations of arthroscopy and osteotomy were identified for the treatment of DDH with concomitant hip pathologies such as cam deformities. Finally, six techniques, all of which are modifications to total hip arthroplasty (THA), were identified for the treatment of high-grade DDH. The techniques detailed in this review therefore equip surgeons with the necessary knowledge to improve outcomes in patients with varying degrees of DDH.

## 1. Introduction

A variety of novel techniques to treat developmental dysplasia of the hip (DDH) in adults have been introduced in the last decade and it is our goal to provide an updated and extensive overview of them for patients with borderline to very severe DDH. Previous systematic reviews examined outcomes of standard techniques to treat adult DDH such as the Bernese periacetabular osteotomy (PAO) or evaluated outcomes for treatment of only borderline adult DDH [1,2]. While these previous studies have provided insightful knowledge regarding the treatment of adult patients with DDH, our aim in this review is to present an extensive overview of each novel technique along with its respective out-comes in treating various degrees of DDH. The novel techniques introduced in this review encompass combinations of arthroscopy, osteotomy, and arthroplasty.

Developmental dysplasia of the hip (DDH) encompasses a multitude of pathologies involving the acetabulum, and occasionally the proximal femur, such as acetabular dysplasia, hip subluxation, true dislocation of the hip, and hip instability. The structural abnormalities present in both the bones and soft tissues surrounding the hip joint can cause the femoral head to move abnormally within the acetabulum which can lead to increased stress on the acetabular rim. In turn, this increases the risk of chondral degeneration and eventually leads to the development of secondary osteoarthritis if left untreated [3,4]. Acetabular dysplasia specifically is defined as inadequate coverage of the femoral head due to a shallow acetabulum and is the subset of DDH pathologies that is more commonly identified in adolescents and adults [5].

Three commonly used methods to determine the severity of DDH in adult patients, which are also used in this review, include the lateral center-edge angle (LCEA) [3], Crowe’s method [6] and the Hartofilakidis method [7]. In terms of the anteroposterior (AP) pelvic view, the LCEA is used to assess the coverage of the femoral head by the acetabulum with an LCEA of 25°–39° being considered normal, that of 20°–25° being considered borderline, and that of <20° being considered dysplastic [3]. Crowe’s method classifies the degree of dysplasia on a scale of I–IV and it states that a larger distance between the medial head–neck junction of the affected hip and the reference line joining both inferior margins of the acetabulum is correlated with a higher degree of dysplasia [6]. Crowe type I–II DDH is generally considered a mild pathology when compared to Crowe III–IV hips which are much more challenging to treat due to extensive distortions to the native anatomy [8,9]. The Hartofilakidis method classifies DDH severity in adults based on the location of the femoral head relative to the acetabulum. Dysplastic hips (type A) have a femoral head that is not dislocated outside of the acetabulum even though subluxation may be present, and these are considered the least severe. Hips with low dislocation (type B) have a partially dislocated femoral head which articulates with a false acetabulum which also covers the true acetabulum to some degree. Hips with high dislocation (type C) have a completely dislocated femoral head which has migrated superoposterioly and has no articulation to the true acetabulum, and this is considered the most severe type [7,10].

THA remains the main treatment for patients with end-stage osteoarthritis secondary to DDH [11]; however, it can be challenging in the setting of DDH due to the anatomy of the dysplastic hip [12]. For this reason, continuous modifications are made to THA in the treatment of DDH to improve results and make it a less demanding procedure. Osteotomy allows early intervention before THA is indicated, and even delays the need for THA by many years [13]. For borderline dysplastic hips (LCEA of 18°–25°) isolated hip arthroscopy is the recommended surgical treatment. However, periacetabular osteotomy (PAO), with or without arthroscopy, may also be beneficial, especially in hips at the upper end of the LCEA spectrum of 18°–25°. Evidence of significant improvements in PROMs is seen in isolated hip arthroscopy, and this is likely due to the effect of addressing intra-articular pathologies, such as labral tears and femoral cam deformities, rather than postoperative radiographic measurements of dysplasia [14,15]. PAO in borderline dysplasia has also shown significant improvements in PROMs; however, categorization of borderline hips with a LCEA of 18°–25° is overly simplistic as this measurement alone does not take into consideration aspects such as the anterior and posterior head coverage. Additional radiographic measurements such as those of the ACEA, Tönnis acetabular roof angle, the anterior and posterior wall indices, and the femoral epiphyseal acetabular roof (FEAR) index may reveal a much more severe degree of dysplasia than the LCEA criteria suggests [16,17]. Isolated arthroscopy is therefore recommended for patients at the upper end of the LCEA spectrum of 18°–25° whereas PAO is recommended for hips with more severe dysplasia. PAO is a successful intervention for adult DDH with a 20-year 60% survivorship rate [18]. There have been many modifications made to PAO as well as new osteotomy techniques in recent years. However, many authors have noted intra-articular pathologies causing symptoms after PAO that are not limited to femoroacetabular impingement (FAI) and labral tears which require treatment with additional arthroscopy [19,20,21,22,23,24,25].

Treatment options change based on a surgeon’s discretion with respect to correcting the specific pathology of a given patient. It is not feasible to compare procedures to identify a universally optimal approach to treatment. Rather, this paper aims to present and summarize the current novel techniques regarding the surgical treatment of adult DDH to quickly familiarize surgeons with available techniques. We hereby present a review of the most common surgical modalities and their respective clinical outcomes pertaining to the treatment of adult DDH. 

## 2. Materials and Methods 

We followed the guidelines indicated in Preferred Reporting Items for Systematic Reviews and Meta-Analyses (PRISMA). Since we intended to provide an updated review of recent novel techniques for either the femoral and/or acetabular management of adult DDH with PROMs, studies only from 2010 onwards were included. We performed computer systematic literature searches of the Embase and Pubmed databases from 2010 to 2 April 2022. The Embase and Pubmed databases were searched with three different search term criteria. Both databases were first searched with the search terms “hip dysplasia” and “osteotomy” in all fields (title, keywords, abstract, etc.). A second search was conducted with the terms “hip dysplasia” and “arthroscopy” in all fields. A third search was conducted with the terms “hip dysplasia” and “arthroplasty”. Searches were performed in two separate databases and very broad search terms were used to ensure we did not miss any articles presenting novel techniques for the treatment of adult DDH. The abstracts were compiled in a reference management software, Endnote.

**Figure 1:**
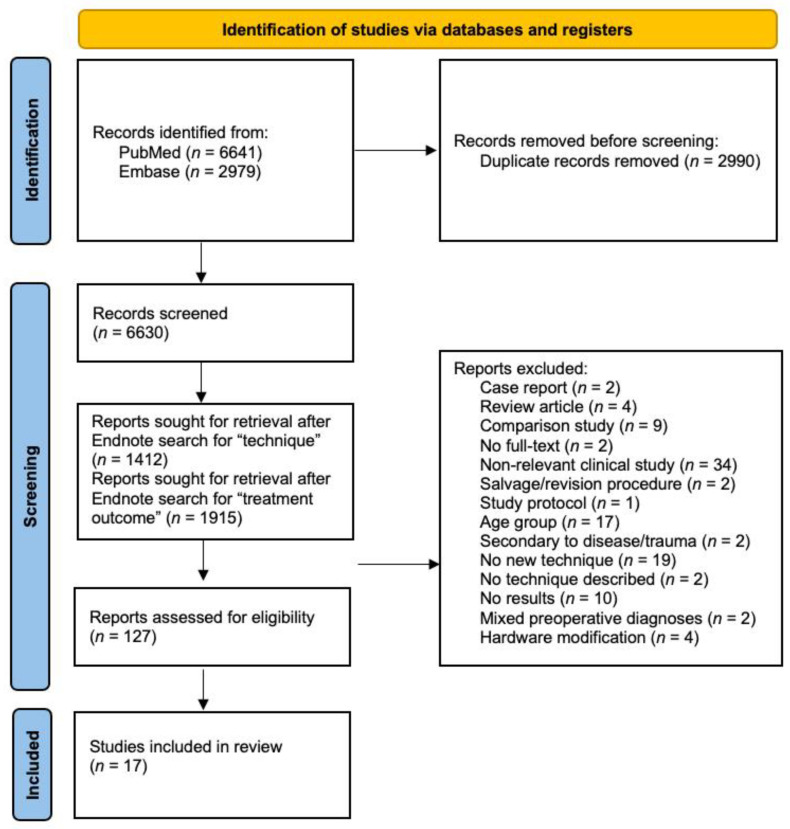
Four-phase PRISMA flow diagram.

Two authors independently performed the study selection. Exclusion criteria included systematic reviews, case reports, letters to the editor, conference abstracts, non-English language studies, studies examining salvage/revision procedures, pediatric studies and studies treating hip dysplasia secondary to other diseases (cerebral palsy, Legg–Calve–Perthes disease, septic arthritis, etc.). Duplicates were removed, narrowing down the list of abstracts to 6,630. An Endnote search was performed for the term “technique” in all terms, resulting in 1412 articles. Abstracts of all these articles were screened, and 57 of them discussed a novel surgical technique for the treatment of adult DDH so they were selected for full-text analysis. An additional Endnote search was performed for the keyword “treatment outcome” resulting in 1915 articles. Abstracts of all these articles were screened, and 89 of them discussed outcomes of surgical treatment for adult DDH so they were also selected for full-text analysis. A four-phase flow diagram of the literature selection was prepared according to the guidelines laid down by PRISMA (Figure 1). This diagram depicts the number of studies identified upon the initial search, the number of duplicate studies between both databases, the keywords used to narrow down our search, and reasons for excluding studies after conducting a full-text analysis of the 127 articles.

## 3. Results

### 3.1. Literature Search and Study Characteristics

The total number of references for each database was as follows. The Embase search returned 2979 abstracts; the PubMed search returned 6641 abstracts. Our electronic database search resulted in 9620 publications for review.

A total of 17 articles with level IV evidence were included in the final analysis [26,27,28,29,30,31,32,33,34,35,36,37,38,39,40,41,42]. The sample size ranged from 11–161 patients (12–200 hips). The follow up time ranged from 1 to 18 years. There were six papers introducing modified techniques for THA, of which two techniques included an additional osteotomy. There were nine papers introducing modified PAOs, of which two techniques included an additional arthroscopy. Two papers discussed modified arthroscopy techniques. The additional techniques introduced are the CU (University of Colorado) PAO, the Birmingham interlocking pelvic osteotomy (BIPO), eccentric rotational acetabular osteotomy (ERAO), reverse (anteverting) periacetabular osteotomy (RPAO), Salter osteotomy, capsular arthroplasty, and endoscopic shelf acetabuloplasty.

Most papers reported outcomes using HHS (12 of 17) with three papers reporting the modified Harris Hip Score (mHHS), two papers reporting the Non-Arthritic Hip Score (NAHS), and one paper additionally reporting the UCLA and Tegner scores. Most studies made the diagnosis of hip dysplasia using the Crowe–Ranawat classification (1), Hartofilakidis classification (2), Tonnis classification (3), and the LCEA angle. A diagnosis of Crowe IV or Hartofilakidis type C hips warranted THA interventions. 

### 3.2. Techniques for Treatment of Borderline or Low-Grade DDH 

While osteotomies and eventually THA are the mainstay treatment for moderate to severe hip dysplasia, low-grade and borderline dysplasia (LCEA between 18°–25°) can be improved via minimally invasive hip arthroscopy as well as a novel technique that incorporates arthroscopy followed by a unique osteotomy [27] (Table 1). 

In 2017, Chandrasekaran et al. presented the arthroscopic technique of labral seal restoration with acetabular rim resection and capsular plication for the treatment of borderline hip dysplasia [26]. The authors of this paper present this technique to overcome the iatrogenic micro-instability and macro-instability associated with performing arthroscopy in dysplastic hips [43,44,45]. Arthroscopy is performed with a standard anterolateral portal, an anterior portal placed under direct visualization, and a distal lateral accessory portal for labral repair. Following diagnostic arthroscopy, concomitant procedures are performed if indicated. In performing capsular plication, the capsule is elevated from the labrum with the use of electrocautery. The preservation of capsular tissue is necessary for later repair. If acetabuloplasty is indicated, very minimal rim resection (2 mm) is performed. To preserve labral tissue, the labrum is not detached from the chondral junction. The labral repair technique (base refixation technique or circumferential suture technique) is chosen based on labral thickness and the quality of tissue. A femoroplasty is performed if a cam deformity is present. The capsule is closed with a suture shuttle technique as described by Chandrasekaran et al. [46]. Capsule closure is completed with four to six sutures via penetration of the acetabular side with a 90-degree SutureLasso while the femoral side of the capsule is penetrated with a sharp bird-beak grasper inferomedially to the acetabular side of the stitch. 

A total of 55 procedures were included in this study, of which 11 had a LCEA of between 18°–20°. Arthroscopic findings included labral tear in 55 procedures (100%), chondral defects at the labral–chondral junction in 48 procedures (87.3%), and a LT tear was found in 56.4% of procedures, with complete disruption being evident in 2 patients. There were statistically significant improvements in all PROMs at the 2-year follow-up: improvements in the mHHS, HOSADL, HOS-SSS, and NAHS were 20.7, 17.5, 27.6, and 20.0, respectively. Six patients required revision surgery (two retorn labrums after a traumatic event, two removals of symptomatic loose chondral bodies, and two iliopsoas fractional lengthening for symptomatic internal snapping of the hip), of which three had revisions within two years.

In 2019, Mei-Dan et al. introduced the CU (University of Colorado) PAO, A minimally invasive, two-incision, interlocking periacetabular osteotomy [27]. This novel interlocking PAO developed at the University of Colorado combines the “benefits of the Birmingham interlocking pelvic osteotomy (BIPO) and the Ganz PAO”. This technique incorporates the preservation of the posterior column as in the Ganz PAO and the interlocking, two-incision approach of BIPO. A hip arthroscopy is performed on all patients 3–10 days before the PAO. A total of 200 hips from 161 patients were included in this study. The mean follow-up was 20 months (3–33 months). Briefly, 19 hips underwent a concomitant proximal femoral derotational osteotomy. Five revision PAOs were excluded. The mean LCEA improved from 18.8 ± 6.9 preoperatively to 31.5 ± 5.9 at the final follow-up. The mean Tonnis angle improved from 12.0 ± 6.5 preoperatively to 0.6 ± 4.2 at the final follow-up. The mean NAHS improved from 56.0 ± 17.9 preoperatively to 81.2 ± 15.3 at 6 months of follow-up and 87.3 ± 11.9 at 12 months of follow-up. Two hardware failures occurred in the initial development of the technique that required refixation. There was one inadvertent intra-articular osteotomy. Minor complications included lateral femoral cutaneous nerve paresthesia in 130 patients (65%) but this was resolved in 85% of patients in the first 6 months.

### 3.3. Techniques for Treatment of Adult DDH (LCEA < 18)

THA remains the main treatment for patients with end-stage osteoarthritis secondary to DDH [11]. THA can be challenging in the setting of DDH due to the anatomy of the dysplastic hip, including soft tissue retraction, a hypoplastic true acetabulum, a high-riding femur, and a neo acetabulum [12]. For this reason, continuous modifications are made to THA in the treatment of DDH to improve results and make it a less demanding procedure. Osteotomy allows early intervention before THA is indicated, and even delays the need for THA by many years [13]. The gold standard is PAO, first described by Reihnold Ganz [47]. There have been many modifications made to PAO as well as new osteotomy techniques in recent years. The following table outlines various novel techniques in the treatment of moderate to severe DDH (Table 2). 

In 2017, Mei-Dan et al. introduced the Birmingham interlocking pelvic osteotomy for acetabular dysplasia. The authors present a novel triple osteotomy called the Birmingham interlocking pelvic osteotomy (BIPO) [28]. This was originally introduced by Kumar et al. in 1992 in patients with Legg–Calve–Perthes disease [48]. The purpose of developing the BIPO was to improve the safety and reproducibility of pelvic osteotomies and to permit unrestricted postoperative weight bearing with faster recovery. The procedure is broken down into two stages. The first stage is the ischial osteotomy. A posterior mini-incision approach is used. In the second stage, an anterior skin incision is completed as is performed in the Bernese PAO. A total of 116 hips of 100 patients were included in this study. The mean follow-up was 17.5 years. The mean difference from preoperative to postoperative values for the Sourcil angle was 20.6 (18.1–23.0) and the mean difference for LCEA was 30.7 (28.4–33.0). The mean preoperative and postoperative scores were not provided. Only the median scores and mean difference were given. There was a high mean postoperative LCEA score due to intentional overcoverage. In one case, overcoverage caused pathological retroversion which required rim trimming. At the latest follow-up, 38 hips had converted to hip arthroplasty with 34 resurfacing arthoplasties and 4 THAs. Hips not requiring revision had a median OHS of 41 and a median UCLA of 5. Only the first 15 hips had the mean HHS, which improved from a median of 52 preoperatively to a median of 90.5 postoperatively.

Salter osteotomy is a procedure used typically for children between ages 2 and 10 to correct early diagnosed hip dysplasia [49,50]. Schimdutz et al. wanted to assess whether or not Salter osteotomy can correct late-diagnosed hip dysplasia [29]. This surgery was performed as described by Salter in 1978 [51] with a few modifications. Additionally, this is a new technique in the scope of treating adult DDH, making this an important article to include in this review. The following modifications were made:

1. Supine position;
2. Removal of wedge-shaped graft proximal to ASIS;
3. Salter maneuver performed on tilted operating table;
4. Fragment fixation with guide wire;
5. Final acetabular correction in supine position using image intensifier.

A total of 49 hips from 45 patients were included in this study. The mean follow-up was 6.7 ± 2.7 years. The mean LCEA improved from 15.5 ± 9.3 preoperatively to 35.2 ± 10 postoperatively. The mean acetabular index (AI) decreased from 15.4 ± 6.8 preoperatively to 4.9 ± 6.6 postoperatively. The mean migration percentage improved from 33.2 ± 9.9% preoperatively to 14.4 ± 9.3% postoperatively. Two patients had non-union. Four patients had wound impairment due to metal rods. Two patients had deep wound infections. Three patients had nerve injury, one of which was not resolved. At 6.7 years of follow-up, no patients that could be contacted had converted to THA.

In 2018, Dienst et al. modified the PAO through a double approach [30]. This modification was made based of the authors’ experience with Tonnis’s triple osteotomy [52]. The goal in this modification is to allow direct vision during the osteotomy of the ischium and the caudal part of the retro acetabular osteotomy. Key modifications are performing the ischial and caudal part of the retroacetabular osteotomy in a “slightly tilted forward” lateral decubitus position and performing the ASIS osteotomy without the exposure of the AIIS, which allows an avoidance of a tenotomy of the rectus femoris tendon. A total of 37 hips in 34 patients were used in this study. The mean follow-up was 20.4 ± 10.3 months. The LCEA changed from 13.2 ± 7.5 degrees preoperatively to 26.5 ± 6.7 degrees. The Tonnis angle reduced from 13.8 ± 6.5 degrees to 3.4 ± 4.4 degrees. At the final follow-up, the mean HHS was 87.6 ± 13.9. A mean preoperative HHS was not provided. There were no major complications. Multiple patients experienced hypothesia of the peroneal nerve, lateral femoral cutaneous nerve, posterior femoral cutaneous nerve, or the pudendal nerve. All cases of hypothesia were resolved over the course of a few weeks up to a maximum of 4 months.

In 2017, Khan et al. introduced a minimally invasive PAO using a modified Smith–Petersen approach [31]. This technique relies upon the usage of specialized osteotomes (Synthes, Salzburg, Austria) and fluoroscopy so as to allow the hip joint capsule to remain unopened. A cohort study was then used to assess compromises in acetabular correction, complication rates, and functional outcomes. In total, 166 hips of 151 patients were included in this study. The mean follow-up was 2.8 years. The mean LCEA improved from 13.4 (13.23–13.57) to 10.1 (9.93–10.27). The mean AI improved from 18.3 (17.2–19.4) to 3.4 (2.59–4.21). There were variable changes in sensation over the distribution of the lateral femoral cutaneous nerve, but these improved drastically over time. One intra-operative crack through the posterior column was noted but did not affect recovery. Stress fractures occurred in 13 hips (7.8%) with 12 fractures in the inferior pubic ramus and one in the posterior column. Conversion to THA occurred in two patients. This was due to the progressive joint space narrowing in one patient as well as pubic non-union and posterior column stress fracture in the other patient. The THA was performed at 2 years and 18 months post-PAO.

Due to the technical difficulties and learning curve associated with the Bernese PAO, many authors [30,53] including Shon et al. [32] have introduced a double approach to PAO. This technique combines the Smith–Petersen and Kocher–Langenbeck approaches. This technique allows a visualization of the posterior column and ischium, whereas the Bernese PAO is performed without direct visualization of these structures. The modified Smith–Petersen technique was used to perform an osteotomy of the pubic bone and ilium, and the Kocher–Langenbeck method was used to perform an osteotomy of the posterior column and ischium. A chest roll positioner was used to rotate the patient between the lateral decubitus position for the posterior incision and the supine position for the anterior incision. A total of 53 hips of 49 patients were included in the study. The average follow-up was 11 years (8–16 years). The average HHS improved from 61.9 before surgery to 91.9 after surgery. Intra-articular osteotomy was observed in two cases due to extension from the osteotomy site. An additional osteotomy was scheduled for one case due to under-correction. Non-union of the pubic bone was observed in three cases. A cross-over sign and ischial spine sign were found in seven cases. An avulsion fracture of the ASIS occurred in one case intra-operatively. No nerve palsies were noted. A 93% survival rate and osteoarthritis progression of 86% was noted at ten years.

In 2021, Mihalič et al. attempted to reintroduce the usage of PAO in Slovenia, originally abandoned in the 1990s due to the steep learning curve and poor midterm outcomes [33]. With many surgeons now taking advantage of intra-operative fluoroscopy to enhance the visualization of the dysplastic hip [47,54]. Mihalič et al. improved on this modification by introducing a electromagnetic navigation (EMN) system and patient-specific templates (PST). The goal was to reduce complications associated with the PAO learning curve and to increase the “accuracy, repeatability, and safety” of the procedure with the following five steps: a CT scan in the DICOM format is uploaded into a medical software application for the creation of a 3D model; the surgeon and a software specialist plan the cuts and acetabular fragment position; the PST is designed to be congruent with the patient’s anatomy and with holes for Kirschner wires; the PST is created with biocompatible plastic; finally, the surgery is performed as described by Ganz et al. [47] and soft tissue exposure is determined as described by Siebenrock et al. [53] with modifications for the PST and EMN system.

The EMN system eliminated the time-consuming and unreliable process of using intra-operative fluoroscopy. The authors go on to compare the acetabular fragment placement accuracy between EMN and fluoroscopy (control). A total of 40 hips from 35 patients were included in this study. The mean follow-up was 2.87 ± 1.13 years for the EMN group (30 hips) and 6.18 ± 0.92 years for the control (fluoroscopy) group (10 hips). Two major complications occurred in the control group (peripheral peroneal nerve dysfunction and popliteal deep vein thrombosis (DVT)) and zero major complications occurred in the EMN group. The only statistically significant difference between the two groups was the average absolute difference in the planned and achieved LCEA and AI, which was 1.2° ± 1.5° and 1.1° ± 2° for the EMN group and 7° ± 6.1° and 6.3° ± 6.3° for the control group (*p* = 0.02; *p* = 0.03). The average HHS value at the final follow-up was 88 ± 12 in the EMN group and 86 ± 14 in the control group (*p* = 0.84). Direct comparison in a patient that underwent both procedures on opposite hips showed that the difference between the planned and achieved LCEA and AI for the EMN side was −0.3° and −0.2°, respectively, while on the control side, the difference between the planned and achieved LCEA and AI was −8.1° and 3.1°, respectively. 

### 3.4. Techniques for Treatment of Adult DDH (LCEA < 18) with Concomitant Hip Pathologies

PAO is a successful intervention for adult DDH with a 20-year 60% survivorship rate [18]. However, many authors have noted intra-articular pathologies causing symptoms after PAO, and the treatment of these pathologies, which includes femoroacetabular impingement and acetabular labral tears, may result in better patient-reported outcomes [34]. PAO with open arthrotomy has been historically favored to treat such pathologies; however, the use of minimally invasive arthroscopy is being explored given the faster recovery time and lower complication rates [36]. The following papers highlight several new modifications to the existing techniques of treating DDH with concomitant intra-articular pathologies (Table 3).

Domb et al.’s early experience with PAO showed a high prevalence of intra-articular abnormalities at the time of PAO [19,25,55]. This led the authors to perform concomitant hip arthroscopies with all PAO procedures at their institution. In this 2015 paper, Domb et al. describe their early experience with this combination of surgical procedures [34]. In this procedure, the arthroscopy is performed prior to the PAO. A traction table is used with the patient supine. Muscle relaxation is also used but stopped during the PAO. A typical arthroscopy is performed with a standard anterolateral portal, a modified anterior portal, and a distal lateral accessory portal. Following diagnostic arthroscopy and treatment, traction is released and the hip is flexed. A femoral osteoplasty is performed using a 5.5 mm round burr. The patient is then transferred to a radiolucent table. The PAO is then performed as modified by Murphy and Millis [55]. A total of 17 patients were included in this study. The mean follow-up was 2.4 years (0.6–3.3 years). Arthroscopic findings included labral repair (12 patients), partial labral debridement (5 patients), iliopsoas fractional lengthening (4 patients), and loose body removal (1 patient). Eight arthroscopic femoral osteoplasty procedures and two open femoral osteoplasty procedures were performed. Three patients underwent microfracture. The mHHS improved from 63.9 preoperatively to 84.1 at the final follow-up (*p* < 0.001); the NAHS improved from 57.7 preoperatively to 79.5 at the final follow-up (*p* < 0.001); the Hip Outcome Score Activities of Daily Living Subscale value improved from 65.4 preoperatively to 80.1 at the final follow-up (*p* < 0.005); and the Hip Outcome Score Sport-Specific Subscale value improved from 37.7 preoperatively to 74.4 at the final follow-up (*p* < 0.001). 

The Visual Analog Scale (VAS) score decreased from 5.6 to 2.6 (*p* < 0.001). Two wound infections occurred, one of which was treated pharmacologically while the second required reoperation. Pulmonary embolism occurred in one patient due to noncompliance with the discontinuation of oral contraceptives. One patient suffered from partial sciatic nerve palsy (resolved postoperatively on day 3) and an intra-operative posterior column fracture.

In 2017, Uchida et al. provided the clinical outcomes for their new shelf acetabuloplasty endoscopic technique with arthroscopic chondrolabral and capsular repair with Cam osteoplasty [35] originally described in 2014 [56]. This technique was introduced as an alternative to PAO or RAO for young athletes. While the results for PAO and RAO are satisfactory for moderate to severe dysplasia, the long rehabilitation period and uncertainty regarding return to sports constitutes the need for alternative treatments for this demographic [34,57]. Cam deformities are common in highly active individuals with hip dysplasia [58,59,60]. Additionally, high stress on a shallow acetabulum can cause labral tears and capsular laxity [60,61,62,63,64]. All these pathologies are addressed with a combination of arthroscopic labral repair, cam osteochondroplasty, capsular plication, and shelf acetabuloplasty in the following sequence: hip arthroscopy as described by Philippon et al. [65], labral repair, traction release and assessment of peripheral compartment for cam lesion, followed by Cam osteochondroplasty using a motorized round burr, capsular plication through the MAP with the hip at 40 degrees of flexion, and finally shelf acetabuloplasty.

Arthroscopic findings included labral tear, ligamentum teres injury and cartilage damage of the femoral head and/or acetabular rim. Two patients reported an increase in UCLA-AS above the preinjury level. A total of 29 of 32 patients returned to sports. The three patients who did not return to sports did not do so due to knee osteoarthritis, shoulder instability, and choice, respectively. The average time for the return to sports was 9 ± 3.5 months. Two patients experienced transient lateral femoral cutaneous nerve neuropraxia. One patient had a fracture of the shelf graft due to returning to sports without physician permission. 

In 2020, Cho et al. presented the long-term results of periacetabular rotational osteotomy (PARO) used concomitantly with arthroscopy [36], a technique they originally described in 2011 [66]. The reason for designing this technique was the frequent reports of the disadvantages of arthrotomy in the treatment of intra-articular pathologies of the dysplastic hip [67,68,69,70], with expanding indications for the usage of arthroscopy for the dysplastic hip [71]. In this procedure, the osteotomy is performed before the arthroscopy. With the patient in the lateral decubitus position, a curvilinear incision is made 2 cm below the ASIS to 2 cm below the greater trochanter, ending 5 cm below the PSIS. Anterior dissection is performed between the gluteus medius and TFL while a posterior dissection is achieved by splitting the gluteus maximus muscle fibers at the posterior border of the gluteus medius. After the osteotomy of the greater trochanter, arthroscopy is performed while applying manual traction. After addressing any intra-articular pathologies, the surgeon moves on to the PAO. The osteotomy is performed with “specially designed curved osteotomes under image-intensifier control”.

A total of 39 hips of 36 patients were included in this study. The mean follow-up was 12.8 ± 1.7 years (153.5 ± 20.6; range, 121.8–188.5 months). In total, 39 labral tears were found amongst 39 patients; 15 were degenerative (38.4%), 3 were flap (7.7%), 2 were radial (5.1%), 1 was longitudinal (2.6%), 2 was complex (5.1%), 12 was fibrillar (30.8%), and 4 were intact (10.3%). Various chondral lesions were also identified, including 16 Grade 0 acetabular lesions, 15 Grade 0 femoral lesions, 11 Grade 1 acetabular lesions, 10 Grade 1 femoral lesions, 4 Grade 2 acetabular lesions, 8 Grade 2 femoral lesions, 8 Grade 3 acetabular lesions, and 6 Grade 3 femoral lesions. The average HHS improved from 72 (60–83) preoperatively to 90 (68–100) at the latest follow-up (*p* < 0.001). Complications included two stable, nondisplaced fractures of the posterior column intra-operatively, postoperative osteonecrosis of the femoral head in one hip, one DVT, and one heterotopic ossification around the greater trochanter. Only one hip (2.6%) underwent conversion to THA 7.8 years postoperatively.

### 3.5. Techniques for Treatment of Crowe III–IV or Hartofilakidis Type C Hips

Crowe’s method of determining the severity of acetabular dysplasia is the most commonly used one for adult patients. Crowe type I–II DDH is generally considered a mild pathology when compared to Crowe III–IV hips which are much more challenging to treat due to extensive distortions to the native anatomy [8,9]. The Hartofilakidis method is also commonly used to classify DDH severity in adults and is based on the location of the femoral head relative to the acetabulum. Hips with high dislocation (type C) have a completely dislocated femoral head which migrates superoposterioly and has no articulation of the true acetabulum, and this is considered the most severe type [7,10]. In Crowe IV or Hartofilakidis type C hips, THA is often necessary early in life. Challenges include a high hip center, abnormalities in femoral and acetabular anatomy, and soft tissue contractures, and thus require additional osteotomy [72,73,74,75,76,77]. Benefits include decreased LLD, restoration of the hip center without stretching the sciatic nerve, correcting femoral anteversion, and restoring abductor mechanisms [72,74,76,78,79] (Table 4). 

In 2015, Binazzi et al. introduced a new THA technique for treating Crowe IV/Hartofilakidis type C DDH that they have been using and modifying since 1994 [37]. This technique allows the avoidance of the use of subtrochanteric osteotomy by utilizing a two-stage technique of progressively lowering the femur first, and then performing THA. By avoiding osteotomy, patients can potentially gain full limb symmetry and not rely on heel pads or shoe lifts. A total of 11 patients and 12 hips were included in this study. The mean follow-up was 11 years. One patient required revision 5 years after the initial surgery due to infection. The other 11 hips had a mean HHS improvement from 35 ± 5 points preoperatively to 85 ± 5 points at the final follow-up. For the nine unilateral, unrevised THA patients, leg length discrepancy (LLD) was improved from a mean of 5.7 ± 1.1 cm preoperatively to −0.3 ± 0.6 cm postoperatively.

In 2018, Montalti et al. developed a THA approach for Crowe III and IV hips that overcomes the issues associated with non-union after femoral osteotomy and with anatomic cup placement in the true acetabulum. This approach is based on a “high center of rotation, a specific implant, and no femoral shortening osteotomy”. [38] The aim of this THA is to have a high cup placement without lateralization of the acetabular component. The mean HHS increased from 35.7 ± 10.4 preoperatively to 82.8 ± 9.5 at the final follow-up. No clinical difference was noted between Crowe III and IV THAs. Survival rate was 90.5%, with five revisions being required. There were three complications noted: one traumatic dislocation and two sciatic nerve palsies. 

In 2018, Li et al. introduced a new THA technique for Crowe III and IV hips that allows the avoidance of femoral shortening and relies on direct leverage to the shoulder of the femoral stem for “rapid, safe, and easy” reduction. A total of 82 hips were included in this study [39]. The mean follow-up was 5.1 years. The mean HHS increased from 42.1 (24–71) preoperatively to 89.9 (76–100) at the final follow-up. Preoperative Trendelenburg gait was positive in 42 hips preoperatively, but positive in only two hips at the final follow-up. LLD improved by 3.0 cm (1.1–5.5) and 2.5 cm (1.1–3.5) in Crowe III hips and 3.6 cm (1.9–5.5) in Crowe IV hips. The average LLD at the final follow-up was 0.43 cm (SD 0.5). There were complications in 3 out of 33 hips (fracture, dislocation, and femoral nerve palsy).

In 2020, Kayaalp et al. proposed addressing the issue of instability at the osteotomy site associated with transverse shortening osteotomy by using a Zweymuller rectangular femoral stem [41]. The authors hypothesized that the rectangular femoral stem can be used to overcome the issues of instability due to two reasons: the fit-without-fill principle, “by preserving bone stock and obtaining a biological healing process in highly dysmorphic proximal femurs” and the four-point anchorage to the bone on the axial plane. This will thus prevent the need for a graft or additional osteosynthesis. A total of 50 hips of 41 patients were included in this study. The mean follow-up was 41.6 months. The mean HHS improved from 45 ± 14 preoperatively to 92 ± 7.8 postoperatively. The mean VAS scores improved from 8.3 ± 1.7 preoperatively to 1 ± 0.9 postoperatively. The mean LLD improved from 2.9 ± 2.5 cm preoperatively to 0.8 ± 0.6 postoperatively. The mean stem subsidence was 1.7 ± 1.2 mm at six months and 2.1 ± 1.4 mm at the final follow-up. A Trendelenburg sign was present in all patients preoperatively but only in two patients postoperatively. Non-union occurred in one patient due to dislocation after a fall. Union occurred after a revision stem in this patient. Intra-operative fractures occurred in 14% of patients.

Wu et al. wanted to address the difficulty of implantation associated with highly dysplastic proximal femurs in DDH [42]. Of the many barriers associated with highly dysplastic femurs, decreased canal size and a thinner cortex are two issues that result in fracture and poor implantation. In 2020, Wu et al. published a modified proximal femoral reconstruction (PFR) technique that allows surgeons to expand the canal volume seamlessly and reduce the femur length at the surgeon’s discretion. This technique provides comparable results to those of subtrochanteric transverse osteotomy. A total of 26 hips from 24 patients with Crowe III–IV DDH were included in this study. Follow-up was at 3 and 12 months. The mean HHS improved from 33.48 ± 9.06 preoperatively to 84.61 ± 4.78 immediately postoperation and 90.84 ± 4.96 at 3 months. The VAS score was 6.92 ± 0.93 preoperatively, which changed to 1.19 ± 0.80 at 12 months of follow-up. Lower limb discrepancy decreased from 5.34 ± 1.96 cm preoperatively to 1.02 ± 0.77 cm postoperatively. At the last follow-up, there were no cases of non-union or prosthesis loosening. The average union time was 4.35 ± 1.24 months. Complications included four patients developing intermuscular vein thrombosis and one patient having a dislocation at 1 month postoperation due to a fall. 

## 4. Discussion

Depending on severity of dysplasia and the presence of intra-articular pathologies, choosing the optimal surgical approach can be a challenge. Factors such as recovery times, complications and PROMs should be considered. This paper comprehensively and concisely summarizes several novel techniques used in the treatment of DDH and outlines the pertinent considerations that a surgeon should evaluate before a surgical intervention is performed. This review can be consulted to efficiently choose between current techniques in treating DDH.

For patients with borderline hip dysplasia, defined in this case as a LCEA between 18°–25°, two novel techniques for treatment were identified: arthroscopy and the CU PAO. Chandrasekaran et al. [26]. presented a novel method of performing arthroscopic labral seal restoration with minimal acetabular rim resection and capsular plication to overcome iatrogenic the micro-instability and macro-instability commonly associated with performing arthroscopy in dysplastic hips. Importantly, there were no associated complications and no conversions to THA at a minimum of two years of follow-up; however, 6/55 hips required revision surgery. Surgeons should therefore be aware of the potential need for revision surgery when performing arthroscopy on borderline dysplastic hips. The authors of this study recommend this technique for patients in which PAO is too invasive and because of evidence that traditional hip arthroscopy has the potential to exacerbate the instability of the hip [1,15,43,44]. Mei-Dan et al. [27] introduced the CU PAO, a novel technique for borderline dysplastic hips in which routine hip arthroscopy is performed 3–10 days prior to the modified PAO. The authors recommend this technique for patients with substantial hip instability in whom isolated arthroscopy has a high risk of failure. At a two-year follow-up, the CU PAO showed no conversions to THA; however, complications included hardware failure in two patients during the initial development of the technique and an inadvertent intra-articular osteotomy in another patient. Despite three complications, there were significant improvements in the NAHS postoperation.

For less dysplastic hips (Crowe I–III/Hartofilakidis type A–B) and hips that had not progressed to severe osteoarthritis, modified acetabular osteotomies were performed, with the majority of these studies addressing either improving visualization or creating minimally invasive approaches to PAOs. Mei-Dan et al. [28]. created their BIPO, a triple osteotomy, to improve safety, reproducibility, and permit unrestricted postoperative weight bearing. Despite an excellent median HHS of 90.5 at the latest follow-up, 33% of hips converted to THA; however, this may be attributed to the long follow-up of 17.5 years in which case the conversion rate is similar to that of the standard Bernese PAO [80]. Additionally, the Salter osteotomy was introduced into the adult population by Schimdutz et al. [29]; however, due to its limited range of acetabular correction, the Bernese PAO was deemed preferable. Dienst et al. [30] and Shon et al. [32] both introduced modified PAOs to allow better visualization when performing osteotomies. The mean HHS values at the latest follow-up were 87.6 and 91.9, respectively, with major complications of pubic bone non-union [3] and an avulsion fracture of the ASIS [1] occurring in the study by Shon et al. Khan et al. [31] presented a minimally invasive approach to PAO which left the hip joint capsule unopened. Stress fracture was the most common complication [13], with 12 occurring in the inferior pubic ramus. Mihalic et al. [33] built upon intra-operative fluoroscopy by introducing the EMN system and PST which successfully reduced complications associated with the steep learning curve of the Bernese PAO.

In addition to hip dysplasia, in patients with concomitant intra-articular pathologies, such as labral tears and cam deformities, modified combinations of PAO and arthroscopy were identified in this review. Domb et al. [34] described a similar level of complications with improved outcomes in patients undergoing arthroscopy to treat intra-articular pathologies followed by PAO compared to those undergoing isolated PAO. Similarly, Cho et al. [36] described a combined arthroscopy with PARO, allowing the treatment of labral tears and chondral lesions, followed by the osteotomy. Between these two studies, there was only one conversion to THA at 7.8 years in the study by Cho et al., along with excellent improvements in PROs in both studies. Uchida et al. [35] introduced a novel technique combining arthroscopic labral repair, cam osteochondroplasty, capsular plication, and shelf acetabuloplasty. This technique is especially beneficial for athletes for whom PAO alone is not sufficient due to the likely presence of cam deformities, labral tears, and capsular laxity. All athletes in this cohort returned to sports rather quickly at an average of 9 ± 3.5 months, with only three not returning due to non-hip-related reasons. Though a paucity of literature exists that examines the outcome of combined PAO and arthroscopy, these three studies highlight their success, especially in patients with concomitant intra-articular pathologies. Even at a long-term follow-up of nearly 13 years by Cho et al., hip survivorship, defined as not converting to THA, was 97.4%.

For patients with severe dysplasia (Crowe III–IV/Hartofilakidis type C), variations of THA remain the gold standard, while THA with osteotomy has been recently explored as well. Binazzi et al. [37] modified the standard THA by utilizing a two-stage technique prior to THA and avoiding osteotomy. Only one patient underwent revision due to infection. Montalti et al. [38] created a modification to allow high cup placement without acetabular component lateralization. Despite a survival rate of 90.5%, five revisions were required with three severe complications noted. Li et al. [39] modified THA by aiming to avoid femoral shortening, resulting in improved leg length discrepancy along with only three complications. Kayaalp et al. [41] modified THA with a novel osteotomy approach in order to address osteotomy site instability, preventing the need for graft or additional osteosynthesis. The mean HHS improved significantly though intra-operative fractures were noted in 14% of patients. An additional THA plus osteotomy modification was introduced by Wu et al. [42] to address the implantation difficulty associated with highly dysplastic proximal femurs using a proximal femoral reconstruction technique. At the latest follow-up, no cases of non-union or prosthesis loosening were noted, though complications included intermuscular vein thrombosis in four patients and dislocation secondary to a fall in one patient. In all studies, postoperative HHS was categorized as good or excellent as the mean scores ranged from 82.8–92 [80]. To provide a relative comparison, patients undergoing traditional THA report similar postoperative HHS, with mean scores ranging from 75–95.6 [81]. Additionally, complications in these studies were similar to those seen in standard THAs such as wound infections, thromboembolic disease, nerve injury, periprosthetic fracture, and dislocation or recurrent instability [82]. Overall complication rates were, however, lower in these studies with a range of 3.6–21.9% compared to those of standard THA [83].

## 5. Conclusions

The surgical protocols currently used for the treatment of DDH are varied, with sufficient differences in clinical outcomes that warrant a review on the part of all practicing orthopedic surgeons. Rapid familiarization must accompany the advent of new and novel techniques, effectively broadening providers’ knowledge and skill sets with respect to treating DDH. While studies evaluating specific treatment outcomes with regard to current techniques and investigations into the pathogenesis of DDH are numerous, reviews that provide surgeons with an overview of clinical outcomes for different techniques are necessary to maintaining or even elevating the current standard of care. Discrepancies in patient outcomes can be better understood and mitigated with reviews such as this that recapitulate the significant findings of more targeted studies in a comprehensive manner.

The novel techniques presented in this review are categorized by the severity of adult DDH along with presence of concomitant pathologies. Two novel techniques, modified arthroscopy and PAO, were identified for the treatment of borderline or low-grade DDH. Six techniques, most of which were modifications to the Bernese PAO, were identified for the treatment of standard symptomatic DDH. Three techniques which include combinations of arthroscopy and osteotomy were identified for the treatment of DDH with concomitant hip pathologies such as cam deformities. Six techniques, all of which were modifications to THA, were identified for treatment of severe high-grade DDH. The authors of each technique have also made attempts to improve on the various complications and difficulties associated with surgically treating a dysplastic hip. For surgeons looking to adopt new techniques, it is important to identify areas of improvement that can be addressed by these novel methods, as they vary from paper to paper.

## 6. Limitations

This paper has its limitations. While a thorough search was performed with three separate search terms on two separate databases, there is the possibility that some novel techniques were skipped over in the search process. Additionally, the authors of this paper only included articles that included patient follow-ups. There are novel surgical techniques in the literature that do not have patient-reported outcomes available at the moment. Such papers were excluded from the literature search. This paper also does not include a real systematic comparison of the techniques, as the variability of procedures and demographics do not allow us to make direct comparisons. Ultimately, it is difficult to highlight which one of these procedures is superior to the others. Rather, we hope that the information provided allows surgeons to investigate procedures that will assist in improving outcomes in their own patient subsets.

## Figures and Tables

**Table 1:** Study introducing new techniques for treating borderline or low-grade DDH (LCEA between 18°–25°). DDH (developmental dysplasia of the hip); LCEA (lateral-center edge angle); mHHS (modified Harris Hip Score); NAHS (Non Arthritic Hip Score); THA (Total Hip Arthroplasty); LFCN (lateral femoral cutaneous nerve).

Study	Level of Evidence	Sample	Intervention	Preoperative Diagnosis	Outcome Measures	Results (Mean)	Follow-up (Mean)	Conversion to THA Rate	Complications
Chandrasekaran, 2017 [26]	IV	55 hips	Arthroscopy	Borderline DDH (mean LCEA 22.1°)	mHHS	84.4 (range 80.0 to 88.8) (improvement of 20.7)	2 years (minimum)	0% Converted	none
Mei-Dan, 2019 [27]	IV	161 patients (200 hips)	CU (University of Colorado) PAO	DDH (mean LCEA 18.8° ± 6.9)	NAHS	89.4 (improvement of 33.4)	2 years	0% Converted	hardware failures (2) inadvertent intra-articular osteotomy (1) LFCN paresthesia (130 patients)

**Table 2:** Studies introducing new techniques for treating adult DDH. PAO (periacetabular osteotomy); DVT (deep vein thrombosis); IQR (interquartile range);UCLA (University of California Los Angeles Activity Score).

Study	Level of Evidence	Sample	Intervention	Preoperative Diagnosis	Outcome Measures	Results (Mean)	Follow-up (Mean)	Conversion to THA Rate	Complications
Mei-Dan, 2017	IV	100 patients (116 hips)	BIPO	DDH (median LCEA, 15° +/− 4.5)	HHS	Median: 90.5 (IQR: 90 to 100) (improvement of 38.5)	17.5 years	33.04 % Converted	Pulmonary embolism 36 h post-op (1) DVT in opposite limb (2) Non-unions (3) Sciatic nerve palsy (1) LFCN injury (2) Infection (1) Iatrogenic pincer-type femeroacetabular impingement (1)
Schmidutz, 2018	IV	45 patients (49 hips)	Salter Osteotomy	DDH (mean LCEA, 15.5° ± 9.3°)	HHS	85.0 ± 11.8	6.7 ± 2.7 years	0% Converted	Non-union (2) Wound impairment due to metal rods (4) Nerve injury (3)
Dienst, 2018	IV	34 patients (37 hips)	PAO	DDH (mean LCEA, 13.2° ± 7.5)	HHS	87.6 ± 13.9	20.4 ± 10.3 months (median)	0% Converted	No severe complications Hypoesthesia of peroneal, LFCN, posterior femoral cutaneous, and pudendal nerves (multiple)
Khan, 2017	IV	151 patients (166 hips)	PAO	DDH (mean LCEA, 14.2° +/− 1.0)	NAHS, UCLA, Tegner scores	95% CI-NAHS: 58.7 (56.1–63.3) pre-op to 82.9 (80.5–85.3) post-op; UCLA: 4.67 (4.38–4.96) pre-op to 6.83 (6.51–7.16) post-op; Tegner: 2.74 (2.49–2.99) pre-op to 3.78 (3.53–4.03)	2.8 years	0.01% Patients Converted	Variable changes in sensation over LFCN distribution Posterior column intra-op crack (1) -stress fractures (12)
Shon, 2021	IV	49 patients (53 hips)	PAO	DDH (CE angle 2.3 ± 3.3 (0~7))	HHS	91.1 (improvement of 29.2)	11.5 years	7.0% Converted	Non-union of pubic bone (3) Cross-over signs and ischial spine sign (7) Avulsion fracture of ASIS intra-op (1)
Mihalič, 2021	IV	35 patients (40 hips)	PAO (electromagnetic navigation-guided)	DDH (Tönnis grade 0–1) (mean LCEA, 16.35°)	HHS	88 ± 12 (mean improvement of 38)	2.87 ± 1.13 years	0.05% Converted	In control group: peripheral peroneal nerve dysfunction (1) popliteal DVT (1)

**Table 3:** Studies introducing new techniques for treating adult DDH with concomitant hip pathologies.

Study	Level of Evidence	Sample	Intervention	Preoperative Diagnosis	Outcome Measures	Results (Mean)	Follow-up (Mean)	Conversion to THA Rate	Complications
Domb, 2015	IV	17 patients	PAO+ Arthroscopy	DDH (mean LCEA, 11.15° ± 6.96)	mHHS	84.1 (improvement of 20.2)	3 years	0% Converted	Microfracture (3) Wound infection (2) Pulmonary embolism due to medication noncompliance (1) Partial sciatic nerve palsy (1) Intra-op posterior column fracture (1)
Uchida, 2018	IV	32 patients (36 hips)	Endoscopic Shelf Acetabuloplasty	DDH (mean LCEA, 16.0° range 5–24)	mHHS	94.5 ± 8.5 (improvement of 26.1)	32.3 ± 3 months	0.02% Converted	Transient LFCN neuropraxia (2)
Cho, 2020	IV	36 patients (39 hips)	PARO + arthroscopy	DDH (mean LCEA, 8.7° (−9 to 18))	HHS	90 (range 68–100) (improvement of 18)	12.8 ± 1.7 years	0.03% Converted	Stable, nondisplaced fractures of posterior column intra-op (2) Post-op osteonecrosis of femoral head (1) DVT (1) Heterotrophic ossification (1) Conversion to THA 7.8 years post-op

**Table 4:** Studies introducing new techniques for treating adult Crowe III–IV DDH.

Study	Level of Evidence	Sample	Intervention	Preoperative Diagnosis	Outcome Measures	Results (Mean)	Follow-up (Mean)	Conversion to THA Rate	Leg Length Discrepancy (Mean)	Leg Length Improvement (Mean)	Complications
Binazzi, 2015	IV	11 patients (12 hips)	THA	Crowe IV/Hartofilakidis type C DDH. HHS: 35±5	HHS	85 ± 5	11 years	N/A	Pre-Op: 5.7± 1.1 cm. Post-Op: −0.3 ± 0.6 cm	−5.4 cm	Revision required 5 years after initial surgery due to infection (1)
Montalti, 2018	IV	84 hips (80 at final follow-up)	THA	Crowe III–IV DDH; HHS: 35.7 ± 10.4 (range 18.5–46)	HHS	82.8 ± 9.5	15.1 ± 3.1 years	N/A	Crowe III: 19 patients (44%); 0 Longer treated hip: 25 patients (58%), +5 mm (2/12). Shorter treated hip: 4 patients (9%), 4 mm (−2/−8). Crowe IV: 10 patients (27%); 0 Longer treated hip: 12 patients (32%), +3 mm (2/5) Shorter treated hip: 11 patients (30%), −6 mm (−3/−11)	N/A	Traumatic dislocation (1) Sciatic nerve palsy (2)
Li, 2018	IV	74 patients; 82 hips (49 Crowe III; 33 Crowe IV)	THA	Crowe III–IV DDH; HHS: 42.1 (range 24–71)	HHS	89.9 (76–100)	5.1 years	N/A	Average at final follow-up: 0.43 cm (standard deviation, 0.5 cm)	Crowe III- 3.0 cm (1.1–5.5); 2.5 cm (1.1–3.5) Crowe IV- 3.6 cm (1.9–5.5)	Fracture (1) Dislocation (1) Femoral nerve palsy (1)
Tahta, 2020	IV	77 patients (77 hips)	THA	Crowe III–IV DDH; HHS: 53.9 (49–62)	HHS	82.7 (76–95)	38.2 (range 22–52) months	N/A	Pre-op true leg length difference: 4.1 cm (2.9–5.3 cm)	Mean leg lengthening: 3.3 mm (2.4–4.6)	Trendelenburg sign post-op (3) Dislocations (3) Revision due to protrusion development in acetabular cap (1)
Kayaalp, 2020	IV	41 patients (50 hips)	THA + osteotomy	Crowe III-IV DDH; HHS: 45 ± 14	HHS	92 ± 7.8	41.6 months	100% Converted	Pre-op: 2.9 ± 2.5 cm Post-op: 0.8 ± 0.6 cm	−1.9 cm	Trendelenberg sign post-op (2) Non-union which resolved after stem revision due to dislocation after fall (1) Intraop fractures in 14% of pts
Wu, 2020	IV	24 patients (26 hips)	THA + osteotomy	Crowe III–IV DDH; HHS: 33.48 ± 9.06	HHS	84.61 ± 4.78	31.36 ± 10.75 months	100% Converted	Pre-op: 5.34 ± 1.96 cm Post-op: 1.02 ± 0.77 cm	−4.32 cm	Intermuscular vein thrombosis (4) Post-op dislocation due to fall (1)

## Data Availability

Not applicable.
